# Control of Plant Height and Lateral Root Development via Stu-miR156 Regulation of SPL9 Transcription Factor in Potato

**DOI:** 10.3390/plants13050723

**Published:** 2024-03-04

**Authors:** Hongyu Luo, Jiangwei Yang, Shengyan Liu, Shigui Li, Huaijun Si, Ning Zhang

**Affiliations:** 1 State Key Laboratory of Aridland Crop Science, Gansu Agricultural University, Lanzhou 730070, China; luohongyu@gsszlyy.com (H.L.); yjw@gsau.edu.cn (J.Y.); lsy18298300908@163.com (S.L.); lisg@gsau.edu.cn (S.L.); hjsi@gsau.edu.cn (H.S.); 2 College of Life Science and Technology, Gansu Agricultural University, Lanzhou 730070, China; 3 College of Agronomy, Gansu Agricultural University, Lanzhou 730070, China

**Keywords:** miR156, *StSPL9* gene, plant height, lateral root, potato

## Abstract

MicroRNAs (miRNAs) are a class of endogenous, non-coding small-molecule RNAs that usually regulate the expression of target genes at the post-transcriptional level. miR156 is one of a class of evolutionarily highly conserved miRNA families. SQUAMOSA PROMOTER BINDING PROTEIN-LIKE (SPL) transcription factor is one of the target genes that is regulated by miR156. SPL transcription factors are involved in regulating plant growth and development, hormone response, stress response, and photosynthesis. In the present study, transgenic potato plants with overexpressed miR156 were obtained via the *Agrobacterium*-mediated transformation method. The results showed that the expression levels of the target gene, *StSPL9*, were all downregulated in the transgenic plants with overexpressed Stu-miR156. Compared with those of the control plants, the plant height and root length of the transgenic plants were significantly decreased, while the number of lateral roots was significantly increased. These results revealed that the miR156/SPLs module was involved in regulating potato plant height and root growth.

## 1. Introduction

MicroRNA is a type of endogenous non-coding single-stranded RNA, generally 20–24 nucleotides in length, which regulates gene expression at the post-transcriptional level through complementary pairing with the target gene mRNA [1]. miRNA was first discovered in *Caenorhabditis elegans* [2]. miRNAs in plants were first obtained from *Arabidopsis* small-molecule libraries [3]. A large number of plant miRNAs have been isolated in *Arabidopsis* [4], rice [5,6], maize [7], wheat [8], and potato [9], and more and more miRNAs have been identified in plants. In total, 38,589 miRNA precursor sequences and 48,885 mature sequences of 217 species were recorded in the miRNA database miRBase (http://mirbase.org/) (Release 22.1: October 2018).

The expression of miRNA is necessary for normal plant growth and development. miRNA is widely involved in plant growth and development, signal transduction, and stress response through negatively regulated transcription factors. Therefore, studying the target genes regulated by miRNA can better reveal the mechanism of miRNA in plant growth and development, and the plant response to stress. At present, the function of miRNAs is studied by increasing or decreasing the expression level of miRNAs in plants. The discovered miRNAs play an important role in the biosynthesis of many plants, and are involved in root [10], stem and leaf [11], and flower development [12], signal transduction, and stress response in different environments. For example, the expression of miR159 is regulated by gibberellin, the target gene of which has GAMYB-like transcription factors the activate the LEAYF gene in meristems of flower organs, thereby regulating flowering time and anther development in short-day plants [13]. miR160 negatively regulates ARF10, ARF16, and ARF17, which are involved in seed germination and post-embryonic development [14]. miR172 controls plant flowering time and floral organ development by regulating the tagged transcription factor targets of early activation (TOE1 and TOE2) [15]. miR164 regulates plant lateral organ development and nutrient organ growth by modulating the NAC family of transcription factors [16]. Overexpression of miR165 downregulates the target gene homeodomain–leucine zipper (*HD-ZIP* III), leading to leaf curling, infertility, and the inhibition of apical meristem growth in plants. The regulation of the *HD-ZIP* III gene by miR165/166 is mediated by tissue-specific expression and different cleavages of target mRNA [17]. In addition, the normal growth and development of plants cannot be separated from hormonal responses, and many signal transduction-related genes are regulated by miRNA. Therefore, miRNA plays important functions in responding to plant hormone response and signal transduction.

miR156 is one of the most abundant and evolutionarily conserved miRNA families in plants, and has 12 family members including miR156a, miR156b, miR156c, and others. Its main target gene is the SQUAMOSA PROMOTER BINDING PROTEIN-LIKE (SPL) gene [18]. Members of the SPL gene family have a conserved 76-amino-acid squamous promoter binding protein (SBP) domain, which is composed of a novel zinc finger with two zinc ion binding sites and can recognize GTAC as the core sequence DNA *cis*-element [19]. As a plant-specific family of transcription factors, SPL is involved in metabolic processes such as plant growth and development, hormone response, stress response, and photosynthesis. Research on a model plant found that the expression of plant SPL genes is regulated by miR156. For example, there are 19 SPL genes in rice, of which 11 are targeted by miR156. Studies on *Arabidopsis* have found that miR156 targets 11 of the 17 SPL genes [20]. According to their functions, they are divided into three categories: the first category has the members SPL2, SPL9, SPL10, SPL11, SPL13, and SPL15, which mainly regulate the transition from the seedling stage to the mature stage. Among them, SPL9 and SPL10 are involved in plant lateral root development, and they also directly bind the miR172b promoter to regulate miR172 expression. The second category of members, SPL3, SPL4, and SPL5, participate in the promotion of the flower meristem identification transition. The third-category member SPL6 may participate in regulating some physiological processes, but its exact function has not been fully elucidated [21]. Numerous studies have shown that miR156 is widely involved in the regulation of leaf shape, lateral root development, flowering time, and the response to drought and other stresses in different species such as *Arabidopsis*, rice, and tomato. Sneha Bhogale et al. (2014) found that overexpression of miR156 in potato not only regulated plants to produce more branches, leaves, and trichomes, but also delayed flowering or even led to non-flowering by regulating some members of the SPL family [22].

Lateral roots are the main components of plant roots, which have the function of absorbing and transporting nutrients and water, as well as the function of stabilizing plants. Recent studies have found that lateral root development is affected by phosphorus stress [23,24], ethylene [25,26], auxin [27], and strigolactone [28]. miR156 regulates the *SPL* gene to inhibit adventitious root development, and the number of adventitious roots declines as plants grow [22]. In Arabidopsis, it was found that *SPL10* is involved in the inhibition of lateral root growth and development through the regulation of the AGAMOUS-like MADS-box protein 79 (AGL79) transcription factor, that the *SPL10* gene and the AGL79 transcription factor were significantly downregulated in miR156-overexpressing plants, and that the transgenic plants produced more lateral roots [19]. Niu et al. [18] found that Arabidopsis miR156 is differentially expressed in specific cells and tissues of lateral roots, and regulates *SPL3*, *SPL9*, and *SPL10* genes to inhibit lateral root growth by responding to the growth hormone signaling pathway.

Potato (*Solanum tuberosum* L.) is an important agricultural resource and material for many industrial processes worldwide. Drought and saline stress have severe effects on potato growth, yield, and quality. During long-term evolution, plants usually defend themselves against changes in the external environment by enhancing or reducing the expression of relevant genes. Since potato is an autotetraploid crop and reproduces asexually, genetic segregation is complex and highly susceptible to genetic homogeneity. Therefore, it is particularly important to genetically improve potato varieties and study the molecular mechanisms of growth, development, and stress resistance. Currently, it has been reported that miR156 and its target genes are indispensable throughout the plant life cycle. Numerous studies have shown that miR156 is most highly expressed in the seedling stage of plants, and is involved in regulating the transition from the seedling stage to the maturity, with a gradual decrease in expression. In addition, miR156 mainly regulates the target gene *SPL*, which is involved in the growth and development of plant leaves and lateral roots, flowering time and flower development, fruit development, and response to stress. Therefore, it is of great significance to study the functions of miR156 and the target gene *SPL* in the growth and development of potato to cultivate new potato varieties.

In the present research, based on the conserved miR156/SPL regulation module, artificial miRNA technology was used to construct the Stu-miR156 overexpression vector, and transgenic potato plants were obtained. The expression level of the Stu-miR156 target gene, *StSPL9*, was analyzed in different tissues of the transgenic and wild-type test tube seedlings, and the number of lateral roots and the length of the lateral roots in the transgenic plants were statistically analyzed to further elucidate the function of the Stu-miR156/*StSPL9* module in regulating plant growth and development in the pot.

## 2. Results

### 2.1. Analysis of Stu-miR156 Secondary Structure and Target Gene

**Figure 1:**
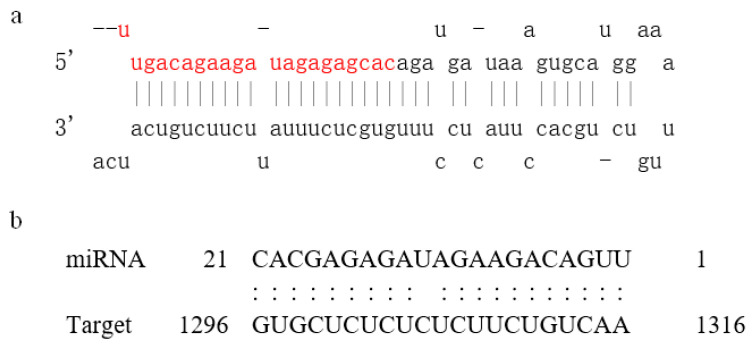
Predicted hairpin secondary structure of Stu-miR156 and its binding site for the target gene *SPL9*. (**a**) Hairpin secondary structures of Stu-miR156 in potato. Mature miRNA sequences are in red. (**b**) Analysis of the binding site of Stu-miR156 for the target gene *SPL9*. Target gene binding site from 1296 to 1316 nucleotides.

Using miRBase (http://www.mirbase.org/) (Release 22.1: October 2018) to predict the secondary structure of Stu-miR156, the mature sequence of miR156 was marked in red (Figure 1). The target gene online prediction software psRNATarget (http://www.zhaolab.org/psRNATarget/) (2017 Update) was used to analyze the binding site of Stu-miR156 to the target gene *SPL9*. The results showed that it was targeted to bind the *SPL9* gene at 1296–1316 bp (Figure 1). We investigated whether or not the predicted target gene *StSPL9* is degraded by miR159, and further validated this through transgenic methods.

### 2.2. Construction of Stu-miR156 Overexpression Vector

**Figure 2:**
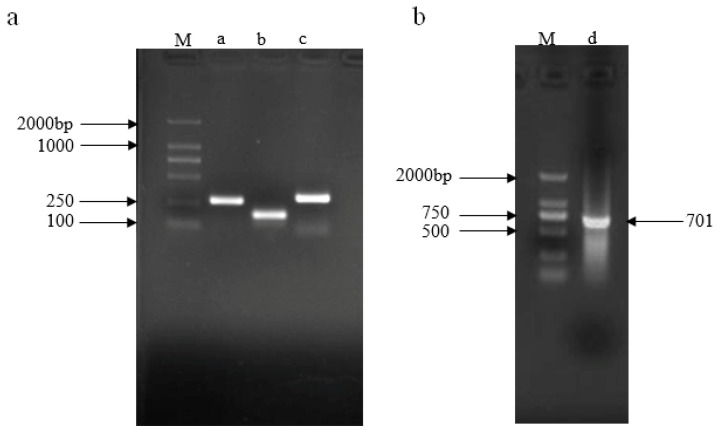
PCR product of amiRNA156 precursor fragment. (**a**) Amplification of a, b, and c fragments; (**b**) amplification of d fragment. M: DL2000 marker (Tiangen Biotech, Beijing, China).

The plasmid pRS300 was used as a template to amplify the 5′ arm (fragment a), the central loop (fragment b), and the 3′ arm (fragment c), the lengths of which were 272, 171, and 298 bp, respectively, using overlapping PCR. The recovered products were used as templates. Primers A and B amplified fragment d, which was 701 bp in length (Figure 2).

### 2.3. Assay of the Putative Potato Transgenic Plants

**Figure 3:**
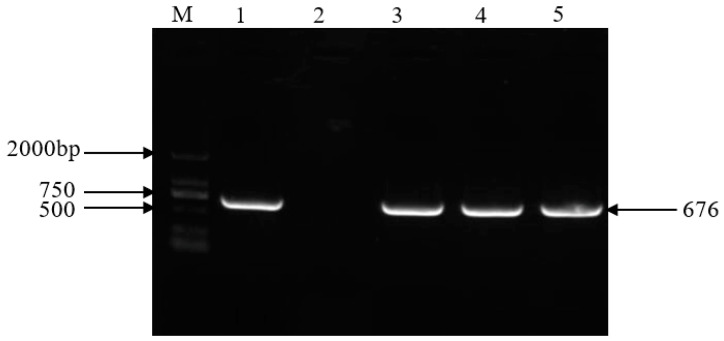
PCR detection of *NPT II* gene in the transgenic plants. M: DL2000 marker (Tiangen Biotech, Beijing, China). 1. Vector plasmid. 2. Non-transgenic plants. 3–5. Transgenic plant lines.

The putative potato transgenic plants initially selected were tested via PCR to amplify the *NPT* II gene (Figure 3). The results showed that three transgenic lines amplified the target fragment (676 bp) measuring the expected size, and non-transgenic plants did not. The results preliminarily showed that the overexpression cassata of Stu-miR156 had been transformed into potato.

### 2.4. Analysis of miR156 and StSPL9 Expression via qRT-PCR

**Figure 4:**
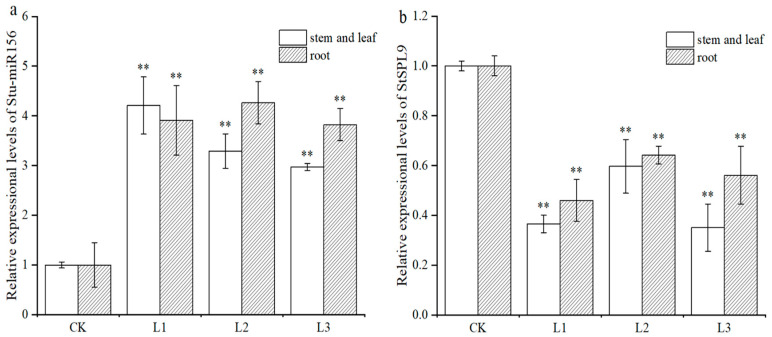
Expressional levels of Stu-miR156 and its target gene *StSPL9* in the transgenic and non-transgenic potato plants. (**a**) qRT-PCR analysis of Stu-miR156. (**b**) qRT-PCR analysis of *StSPL9*. CK. The non-transgenic potato as a negative control. L1–L3. The transgenic potato plant lines transformed with the vector pCPB121-miR156. Data are presented as a mean ± SD (three biological replicates). The asterisks represent significant differences compared with the control (** *p* < 0.01).

The transgenic and non-transgenic potato plants were cultured on MS media containing 2% sucrose for 3 weeks. qRT-PCR was performed to determine the relative expression levels of miR156 and its target gene *StSPL9*, and the correlation between the generation of lateral roots and their expression was further analyzed. The qRT-PCR results showed that miR156 expression in roots, stems, and leaves of the transgenic lines L1, L2, and L3 was upregulated by 2 to 4.4 times compared with that in the non-transgenic plants. Otherwise, the target gene *StSPL9* was downregulated in roots, stems, and leaves of the transgenic lines L1, L2, and L3 by 2.9 to 1.6 times compared with that in the non-transgenic plants. Furthermore, the expression in roots was higher than that in stems and leaves (Figure 4). The results showed that the expression trend of Stu-miR156 and its target gene were opposite to each other, indicating that the *StSPL9* gene was degraded by Stu-miR156, decreasing its expression level.

### 2.5. Effect of Stu-miR156 Overexpression on Potato Morphological Characters

**Figure 5:**
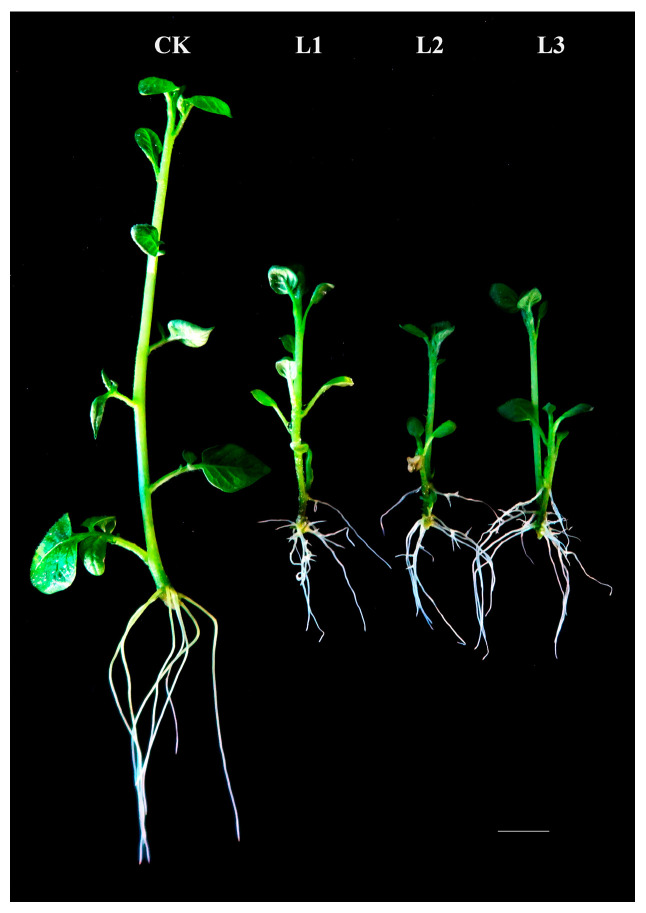
The phenotypic characteristics of the transgenic potato plants. CK. The non-transgenic potato plant as a negative control. L1–L3. The transgenic potato plant lines. The scale bars represent 2 cm.

To study the role of Stu-miR156 in regulating potato morphology, we analyzed the differences in plant height, lateral roots, fresh weight, and root:shoot ratio between the transgenic and non-transgenic plants. The results showed that the number of lateral roots of the transgenic lines with miR156 overexpression increased significantly by 2.8 to 3.6 times compared with that in the non-transgenic plant control, while the mean values of root length, plant height, fresh weight, and root:shoot ratio were all decreased, being 0.29, 0.62, 0.20, and 0.46 times those of WT plants, respectively (Table 1, Figure 5). It is indicated that after the overexpression of the miR156 gene, the growth of the plant is inhibited. It is speculated that the miR156/*StSPL9* module plays an important regulatory role in potato development and growth.

## 3. Discussion

The root system is the basic organ of the plant and consists of the lateral roots and root hairs of the plant. Its growth is affected by plant hormones, transcription factors, and miRNAs [29]. MicroRNA is an important type of gene expression regulator, and plays a very important role in regulating plant morphogenesis and responding to biotic and abiotic stress [4]. Many miRNA target genes encode transcription factors. Therefore, miRNA target genes play an important role in regulating plant morphogenesis through protein–DNA and protein–protein interactions [30,31]. miR160, miR164, and miR165/miR166 all regulate the development of lateral roots through the auxin signaling pathway, nutritional homeostasis, and response to environmental stress [32,33,34]. miR156 is one of the most abundant and evolutionarily highly conserved miRNAs in plants. It is mainly expressed in plant seedlings. It is characterized by increased branching, accelerated leaf budding, and delayed flowering, and its expression level decreases with increasing seedling age [18]. The involvement of miR156 in plant root development has been reported, but there is no specific report on whether or not miRNA156 is involved in potato root development. Therefore, in this study, the precursor of the miR156 gene in potato was cloned from potato ‘Desiree’ as the experimental material (Figure 2), and bioinformatic analysis was carried out to conduct an in-depth study on its function. Many studies have shown that most SPL family genes in plants are the target genes of miR156, and the miR156/SPL regulatory mode has become the hub of regulating plant growth and development [35]. Most members of the SPL family in Arabidopsis [20], rice [36], and other plants have been confirmed to be the target genes of miR156. This study also predicted that the target gene of miR156 is SPL (Figure 1), indicating that miR156-mediated post-transcriptional regulation is conserved in plants. These results also indicate that miR156 is important in regulating the SPL family.

In recent years, with the in-depth study of miRNA, the enhancement and inhibition of target miRNA expression through synthetic mimics of mature miRNA and their inhibitors are gradually being applied; these mimics can effectively enhance and inhibit the expression of target genes, and thus play a role in regulating the level of related genes. This study identified miR156 and its target gene SPL9’s transcription factor in potato. Artificial microRNA technology was used to construct the Stu-miR156 overexpression vector, and transgenic plants with Stu-miR156 overexpression were obtained. The results showed that Stu-miR156 was expressed differently in different tissues (Figure 4), indicating that miR156 played different functions in different tissues of potato. The expression levels of the Stu-miR156 vector and *StSPL9* target gene were analyzed in different tissues of the transgenic and wild-type test tube seedlings (Figure 4). The results showed that the expression trends of Stu-miR156 and its target gene were opposite to each other, indicating that the *StSPL9* gene was degraded by Stu-miR156, decreasing its expression level. The expression of the Stu-miR156 target gene *StSPL9* was downregulated in transgenic potato roots, stems, and leaves, indicating that Stu-miR156 and its target genes are widely involved in regulating plant morphology (Figure 5), which is consistent with the previous study [18]. miR156 is necessary and sufficient for juvenile expression, and it is suggested that it works by inhibiting the expression of SPL genes with different developmental functions [20,37]. In *Arabidopsis*, it was found that *SPL9* is specifically expressed in the lateral root meristematic tissue region, whereas *SPL10* is expressed at the lateral root initiation site and regulates the AGAMOUS-like MADS-box protein 79 (AGL79) transcription factor involved in the inhibition of lateral root growth and development. The *SPL10* gene and AGL79 transcription factor were significantly downregulated in overexpressed miR156 plants, and transgenic plants produced more lateral roots [19]. The analysis of the number of lateral roots showed that the number of lateral roots of the transgenic plants with miR156 overexpression significantly increased, which indicated that the regulation mode of miR156/SPL is involved in regulating the development of lateral roots in potato. Studies have shown that over-expression of miR156a can lead to increased primary root growth and promote the development of lateral roots. SPL3, SPL9, and SPL10 positively regulate miR156 expression in plant lateral root development. The expression of transgenes SPL3, SPL9, and SPL10 against miR156 can affect leaf development in different ways [12]. SPL10 is highly expressed at the starting site of the lateral roots, while SPL9 is specifically expressed in the meristem region [18]. Gou et al. [38] revealed that miR156-targeted regulation of *SPL9* prevents the expression of anthocyanin biosynthesis genes in Arabidopsis thaliana, thereby negatively regulating anthocyanin accumulation, revealing that *SPL9* regulates the transition from the seedling to flowering stage in plants. In the experiment, the analysis of the root length and plant height of the transgenic plants showed that growth of the transgenic plants with miR156 overexpression was significantly inhibited, and root length and plant height were significantly decreased. Therefore, the regulation mode of Stu-miR156 targeting SPL transcription factors plays an important regulatory role in potato development and growth. This study lays the foundation for the further in-depth elucidation of the involvement of Stu-miR156 in the regulation of potato growth and development. Since one miRNA can degrade multiple target genes, and a target gene can also be degraded by multiple miRNAs, it is not a one-to-one relationship but involves complex network regulation. This study can only conclude that Stu-miR156 can degrade the *StSPL9* gene and participate in plant growth and development. Further research is needed to determine whether or not and which other genes co-regulate this phenotype.

## 4. Materials and Methods

### 4.1. Plant Materials

Potato (*Solanum tuberosum* L.) cultivar ‘Desiree’ was used as an experimental material. The potato plantlets were propagated on MS media containing 2% sucrose and 0.45% agar, and cultured under a 16/8 h light/dark cycle at a temperature of 25 ± 2 °C.

### 4.2. Bioinformatics and Software

The Stu-miR156 mature sequence and its secondary structure were obtained from the miRNA miRbase (http://www.mirbase.org/) (Release 22.1: October 2018). Using the WMD3 website (http://wmd3.weigelworld.org/) and inputting the mature sequence of Stu-miR156 on the Oligo page, primers I, II, III, IV, A, and B were designed for its cloning. The target gene of Stu-miR156 was predicted using online prediction software (http://www.zhaolab.org/psRNATarget/ (2017 Update), and the mature body sequence of Stu-miR156 was input in the ‘Submit small RNAs’ page for prediction.

### 4.3. Amplification of miR156 Precursor Fragment and Construction of Cloning Vector

**Figure 6:**
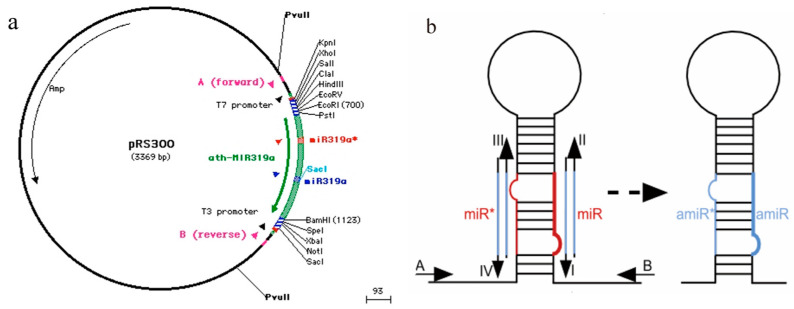
Amplification of miR156 precursor fragment and construction of cloning vector. (**a**) Schematic diagram of plasmid pRS300, which contains the endogenous *Arabidopsis* miR319a precursor in a pBluescript SK backbone and does not contain any promoter. This only serves as a template for PCR directed mutagenesis using oligonucleotides that can be obtained from the WMD webpage (http://wmd2.weigelworld.org) and accessed on 18 August 2019. (**b**) Amplification diagram of precursor sequences of amiRNAs using overlapping PCR. I. miRNA forward primer; II. miRNA reverse primer; III. miRNA* forward primer; IV. miRNA* reverse primer. Oligos I, II, III and IV were replaced with the corresponding amiRNA by the miR319a sequence. “miR*” represents the known miRNA, and “amiR*” represents the corresponding artificially designed miRNA.

Plasmid pRS300 was used as a template, and 20 nucleotides of endogenous miRNA319a in plasmid pRS300 were replaced with Stu-miR156 mature sequences using overlapping PCR. The 5′ arm (fragment a), the central loop (fragment b), and the 3′ arm (fragment c) were amplified. Products a, b, and c were purified and recovered via 2% agarose gel electrophoresis. Fragments a, b, and c were used as templates, and fragment d was amplified using A and B primers (Table 2, Figure 6). Fragment d was ligated into the pMD18-T vector, sequenced, and named pMD-amiR156.

### 4.4. Construction of Plant Expression Vector

The cloning plasmid pMD-amiR156 and expression vector pCPB121 were digested using double-restriction endonucleases *Sac* I and *Kpn* I, respectively. The abovementioned digested products were electrophoresed on 2% agarose gel to recover the target fragments, which were ligated with T_4_ DNA ligase at 16 °C for 30 min, and then transformed into *Escherichia coli* competent cells. The positive clones were selected for the extraction of the plasmid, and *Sac* I and *Kpn* I were used for double-enzyme digestion verification. The constructed expression vector was named pCPB121-miR156 and then transferred into *Agrobacterium tumefaciens* LBA4404 competent cells for the genetic transformation of potato.

### 4.5. Potato Transformation and Identification of the Transgenic Plants

The following method was used for *Agrobacterium*-mediated potato transformation [34]. Three-week-old potato in vitro plantlets were cut into 1 cm stem segments, which were infected with *Agrobacterium* (OD_600_ = 0.5) for 7 min, and the excess bacterial solution was blotted dry with sterile filter paper and then put into 1/2 MS solid media to be cultivated at 25 °C for 48 h in the dark. After co-cultivation, we transferred the stems into callus media (1/2 MS + 5 mg/L NAA + 0.1 mg/L 6-BA+ 0.05 mg/mL kanamycin) and cultured them at 23 °C under a 2000 lx light intensity for 7 days, then transferred them into differentiation media (1/2 MS + 20 μg/L GA3 + 20 μg/L NAA + 2 mg/L zeatin + 0.05 mg/mL kanamycin), replacing them with new media every 10 days until new shoots grew from the stem segment; then, they were put into the rooting media (MS + 75 mg/L kanamycin + 200 mg/L carbenicillin) for screening. After about 2 weeks, the rooted shoots were identified as putative transgenic plantlets for further identification. The hexadecyl trimethyl ammonium bromide (CTAB) method was used to extract the genomic DNA of the putative transgenic plants and of the non-transgenic plants as a negative control. The identification of the transgenic plants was performed using the neomycin phosphate transferase (*NPT II*) gene of a pair of primers (Table 3), and the expected fragment size was 676 bp. The PCR reaction conditions were as follows: incubation at 94 °C for 3 min, followed by 35 cycles of 94 °C for 45 s, 60 °C for 45 s, and 72 °C for 60 s, anfinally extended to 72 °C for 10 min. The PCR products were separated via 1% agarose gel electrophoresis.

### 4.6. Analysis of the Transgenic Potato Plants via qRT-PCR

Roots, leaves, and stems of the 3-week-old non-transgenic plants and transgenic plants were collected and placed in liquid nitrogen for quick freezing. Plant total RNA was extracted using the AG RNAex Pro RNA extraction kit (AG21101). The cDNA used for the expression analysis of miR156 was synthesized via reverse transcription using the One Step PrimeScript1 miRNA cDNA synthesis kit (TaKaRa), and the 18S rRNA of potato was used as a reference gene. The Evo M-MLV reverse transcription kit (AG11705-S) was used for the quantification of target genes, and the *ef1a* gene was the internal reference gene (Table 3). The expression of the target gene was assayed using SYBR^®^Premix Ex Taq^TM^II (TaKaRa). The PCR reaction conditions were as follows: 95 °C for 10 min; 95 °C for 15 s; 60 °C for 1 min; and 72 °C for 30 s for 40 cycles. The calculation formula was as follows: RQ (relative expression) = 2^−∆∆Ct^, ∆∆Ct = (∆Ct _transgenic_ − ∆Ct *_ef1a_*) − (∆Ct _non-transgenic_ − ∆Ct *_ef1a_*). All samples were analyzed in three biological replicates and three technical replicates. Statistical software SPSS 13.0 was used to analyze the differences in the expressions of the transgenic plants. Data were statistically analyzed via one-way ANOVA and Dunnett’s post hoc test.

### 4.7. Morphological Assay of the Transgenic Potato Plants

The transgenic and non-transgenic potato plants were propagated by sub-culturing them on MS media containing 2% sucrose and culturing them under a 16/8 h light/dark cycle at a temperature of 25 ± 2 °C for two weeks. The following indicators were measured using a spiral micrometer: plant height, root length, and root to shoot ratio. The fresh weight of the plants was weighed using an electronic balance. The experiments were conducted in three biological replicates. Significant differences were assayed using Duncan’s multiple-range test at the 5% probability level.

## 5. Conclusions

The SPL transcription factor is one of the target genes that is regulated by miR156. In this study, the expression levels of the target gene StSPL9 were downregulated in the transgenic plants with overexpressed Stu-miR156. The plant height and root length of the transgenic plants were significantly decreased compared with those of the control plants, while the number of lateral roots were significantly increased. In conclusion, the miR156/SPLs module was involved in regulating the plant height and root growth of potato.

## Figures and Tables

**Table 1:** Phenotypic assay of the transgenic plants.

Plant Lines	Plant Height (mm)	Root Number	Root Length (mm)	Fresh Weight (g)	Root-Shoot Ratio (%)
CK	82.74 ± 2.88 a	10.67 ± 1.70 b	59.38 ± 5.55 a	0.48 ± 0.05 a	72.09 ± 0.09 a
L1	53.00 ± 2.17 b	31.33 ± 5.73 a	18.33 ± 1.32 b	0.10 ± 0.02 b	34.73 ± 0.04 b
L2	45.33 ± 7.95 b	29.67 ± 2.87 a	13.99 ± 3.14 b	0.07 ± 0.03 b	30.75 ± 0.03 b
L3	55.51 ± 10.11 b	38.33 ± 6.94 a	19.16 ± 6.52 b	0.11 ± 0.03 b	34.51 ± 0.05 b

Note: Different letters represent significant differences (*p* < 0.05).

**Table 2:** Overlapping PCR to construct a PCR amplification system for amiRNA156.

PCR Reaction	Forward Primer	Reverse Primer	Template	Product Length (bp)
a	A	IV	pRS300	272
b	III	II	pRS300	171
c	I	B	pRS300	298
d	A	B	a + b + c	701

Note: Reaction b is most likely to be difficult, since less than half of the nucleotides in the oligos are complementary to the template plasmid. The reaction can be improved by increasing the cycle number (we used up to 40 cycles without a dramatic rise in PCR-induced mutations).

**Table 3:** Primer sequences.

Primer Name	Primer Sequences (5′→3′)
A	CTGCAAGGCGATTAAGTTGGGTAAC
B	GCGGATAACAATTTCACACAGGAAACAG
I	GATTGACAGAAGATAGAGAGCACTCTCTCTTTTGTATTCC
II	GAGTGCTCTCTATCTTCTGTCAATCAAAGAGAATCAATGA
III	GAGTACTCTCTATCTACTGTCATTCACAGGTCGTGATATG
IV	GAATGACAGTAGATAGAGAGTACTCTACATATATATTCCT
NPT II-F	GCTATGACTGGGCACAACAG
NPT II-R	ATACCGTAAAGCACGAGGAA
SPL9-F	CCTACTGTTGTTGTTGCTGG
SPL9-R	CCTACGACGCTCATTATGG
Stu-miR156	TTGACAGAAGATAGAGAGCAC
St18S RNA	TTAGAGGAAGGAGAAGTCGTAACAA
ef1a-F	CAAGGATGACCCAGCCAAG
ef1a-R	TTCCTTACCTGAACGCCTGT

## Data Availability

Data are contained within the article.
